# Why Do We Eat Comfort Food? Exploring Expectations Regarding Comfort Food and Their Relationship with Comfort Eating Frequency

**DOI:** 10.3390/nu17142259

**Published:** 2025-07-08

**Authors:** Fei Wu, Lenny R. Vartanian, Kate Faasse

**Affiliations:** School of Psychology, University of New South Wales (UNSW Sydney), Sydney, NSW 2052, Australia; l.vartanian@unsw.edu.au (L.R.V.); k.faasse@unsw.edu.au (K.F.)

**Keywords:** comfort food, expectation, emotions, food choices, eating behavior

## Abstract

**Background/Objectives**: Consuming comfort food is a common experience in daily life, but the underlying motives for engaging in comfort eating remain unclear. This study examined people’s expectations regarding their comfort food and investigated whether these expectations are associated with their frequency of comfort eating. As an exploratory aim, we also examined whether there are gender differences in preference for different categories of comfort food (i.e., sweet or savory) and the frequency of engaging in comfort eating. **Methods**: Through an online survey, participants (*n* = 214) reported their primary comfort food, the frequency of comfort eating in the short term (i.e., the past two weeks), and the general trend over the long term. They also rated statements related to their primary comfort food based on five expectation subscales (i.e., Manage Negative Affect; Pleasurable and Rewarding; Enhances Cognitive Competence; Alleviates Boredom; Positive Feelings). **Results**: Although Pleasurable and Rewarding and Positive Feelings received the strongest level of endorsement, their associations with the frequency-related variables were weak in both correlational and regression analyses. In contrast, Manage Negative Affect, Alleviates Boredom, and Enhances Cognitive Competence were positively associated with all frequency-related variables, with Alleviates Boredom showing the most consistent pattern. There were no significant gender differences in preferences for sweet or savory comfort food, and no significant gender differences in the frequency of eating comfort food. **Conclusions**: These findings suggest people believe they can gain a range of expected benefits from consuming comfort foods and perceive themselves as consuming comfort food primarily for rewarding themselves or gaining positive feelings. However, it is the expectations of managing negative affect, alleviating boredom, and enhancing cognitive competence that motivate them to engage in comfort eating.

## 1. Introduction

Comfort food can be defined as food that provides psychological comfort [1,2]. Comfort foods are often high-calorie foods with high sugar and/or fat content (e.g., [1,3,4,5]). For example, in a study involving residents from the United States and Canada, the most commonly reported comfort foods were chips (23%), ice cream (14%), cookies (12%), candy or chocolate (11%), and pasta or pizza (11%) [2]. Given that comfort foods are often calorically dense yet nutrient poor, frequent consumption of these foods can have implications for overall health. Therefore, it is important to understand the reasons why people eat comfort foods.

Researchers often emphasize the role of comfort foods in providing emotional benefits, particularly when people are experiencing negative moods (e.g., [3,5,6]). However, the findings from this literature are somewhat mixed. In one experimental study, eating chocolate was significantly more effective than drinking water in recovery from a mood decline after watching a sad film clip [7]. Other studies have also shown that consuming comfort food led to mood recovery following a stressor or negative mood induction, but they suggest that the beneficial effects might be short-lived [5], and that mood recovery may not differ from participants who did not consume comfort food [8]. Another study found no effects of eating unhealthy comfort food on stress responses and mood, regardless of whether the food was consumed in anticipation of (but prior to) a stressor or after the stressor [9].

Other studies suggesting that comfort foods can have mood-enhancing effects did not involve the actual consumption of comfort food, but instead appear to reflect participants’ beliefs about the benefits of comfort foods. For example, one study found that viewing or drawing pictures of comfort food enhanced positive mood, especially among individuals with clinical symptoms of depression [10]. Another study examined the impact of writing about one’s experience of eating comfort food and found that, under the belonging threat condition, securely attached individuals (but not insecurely attached individuals) felt less lonely after writing about their experience with comfort food compared to writing about a new food [11]. Because these studies do not involve actual food intake, they most likely reflect how people understand and anticipate the outcomes of comfort eating. In other words, people might have expectations that comfort food could bring psychological benefits.

Whether or not eating comfort foods is an effective way to manage one’s emotions, people still do turn to comfort eating in order to improve their emotional state, possibly because they have developed an expectation that comfort food can provide psychological benefits. There is some preliminary support for this suggestion. For example, in one study, participants completed a questionnaire prior to eating comfort food, and 81% of participants either agreed or strongly agreed with the statement “I am confident that eating this food would make me feel better” [5]. In another study, participants retrospectively recalled that their emotional state significantly improved after eating comfort food [3]. Although the researchers did not discuss these findings in terms of expectations, these statements do suggest that people have certain expectations about comfort food.

Given that comfort foods can be used to help people manage their emotional states, the emotion regulation strategies they typically employ could influence what they seek from comfort food. Previous research has demonstrated gender differences in the use of emotion regulation strategies. For example, women tend to use a wider range of strategies than men [12]. Significant gender differences have been observed in cognitive emotion regulation strategies: women reported greater use of rumination and putting problems into perspective, whereas men reported greater use of blaming others [13]. Moreover, men report more suppression of emotional expression than do women [14]. It is possible that these gender differences in emotion regulation may also be reflected in individuals’ expectations related to comfort foods.

Research in other domains have found that people’s expectations about the outcomes of a behavior can influence their engagement in that behavior. Expectancy Theory [15] indicates that expectancy is the belief in actions or events and their subsequent consequences, which people learned from direct or indirect experiences. These expectations are stored in their memory and influence future behavioral choices [15,16]. In the context of substance use, for example, people’s expectations about the effects of substances such as alcohol, tobacco, and marijuana are associated with their level of substance use [17], but the nature of these associations also depends on the specific expectations assessed [18,19,20,21]. Together, these studies highlight the fact that it is important to consider the different expectations that people have, because some may be more strongly related to behavior than others.

Research on expectations related to eating behavior has mostly been conducted in the context of disordered eating behavior in both clinical and healthy populations. For example, in a clinical population, one study found that individuals with bulimia-spectrum eating disorders who had planned at least one binge-eating episode in the previous 28 days had greater expectations that eating would alleviate boredom or reduce negative affect than did those who did not plan a binge-eating episode [22]. In healthy populations, one study has shown that the expectation that eating can manage negative affect is associated with disordered eating among both men and women, and that the expectations that eating is pleasurable and rewarding is inversely associated with disordered eating among women [23]. Another longitudinal study among middle school girls demonstrated the relationship between expectations and maladaptive eating behaviors. Specifically, the expectation that “eating is pleasurable and rewarding” predicted higher levels of social and celebratory overeating, while the expectation that “eating helps manage negative affect” predicted higher levels of binge-eating [24]. Given the findings from the literature on substance use and disordered eating, it may be possible that the consumption of comfort food is also motivated by various eating expectations, but this has not yet been explored.

The aim of the present study was to investigate people’s expectations regarding comfort food, and to determine whether their expectations relate to the frequency of consuming comfort foods. We hypothesized that greater expectations of the benefits of eating comfort foods would be associated with more frequent comfort food consumption. We also assessed whether different expectations are differentially associated with the frequency of eating comfort foods and explored any potential gender differences in preference for comfort food and frequency of comfort eating.

## 2. Materials and Methods

### 2.1. Participants

A total of 220 participants were recruited via Prolific. The required sample size was calculated using G*Power 3.1. For an *F* test (linear multiple regression with 5 predictors) based on a medium effect size of 0.15, *α* = 0.05, and power = 0.80, the minimum required sample size was 92 participants. Given that this was a novel area of research and we were also exploring potential gender differences, we doubled the sample size. Additionally, we increased the sample size by 15% to account for potential data loss. Therefore, a total of 220 participants were recruited for this study.

The inclusion criteria were “Eat comfort foods at least occasionally”, “Be fluent in English”, and “Be aged 18 or above”. The survey advertisement stated that “We are currently conducting a study to understand people’s views of comfort foods. You should only complete this survey if you at least occasionally eat food for psychological comfort.” No examples of comfort foods were provided to avoid potential bias in which comfort foods participants reported eating. All participants were compensated according to Prolific’s standard rate of £9 per hour for completing the survey. Six participants were excluded from the data because they indicated that they never ate comfort foods, leaving 214 participants for analysis. The sample comprised 107 individuals who identified as female, 106 who identified as male, and 1 who identified as non-binary/third gender. Participants’ mean age was 38.73 years (*SD* = 12.60; range = 18 to 74). The mean body mass index (BMI) was 25.38 kg/m^2^ (*SD* = 6.58). Two participants did not report their weight or height, so their BMI could not be calculated. In terms of ethnicity, 83.6% self-identified as White, 7.5% as Asian, 4.2% as African, 0.9% as Middle Eastern, 0.5% as Indigenous Australian or Torres Strait Islander, and 3.2% as “other” or “prefer not to say”. This study was approved by the university’s ethics committee.

### 2.2. Materials and Procedure

All participants completed the survey online. After providing informed consent, participants completed the following measures in order:

***Identification of Primary Comfort Food.*** At the start of the survey, participants were provided with a definition of comfort food (“food that provides psychological comfort”), and the instructions emphasized that comfort food can be highly individualistic, and that the food that provides comfort for one person might not provide comfort to another person. Following these introductions, participants were asked: “When you think about your own personal comfort foods, what’s the first food that comes to mind?” Their open-ended responses to this question were used as the target food in subsequent questions regarding expectations.

Follow-up questions (presented after they had completed the expectation-related measures) asked participants to indicate all applicable reasons for choosing their specified food as their primary comfort food. Specifically, they were asked, “What is the main reason [participant’s primary comfort food] was the first comfort food that came to mind for you?” Response options included the following: “It’s the comfort food I eat most often”; “It provides me with the strongest sense of comfort”; “The comforting effect of this food lasts the longest”; “It has the strongest sentimental meaning in my life”; “It’s deeply rooted in my cultural or family traditions”; “It comforts me in various situations or moods”; “It’s the easiest comfort food for me to obtain”; and “Other (Please specify)”.

***Expectations regarding Comfort Food.*** Expectations regarding comfort food were measured using items adapted from the Emotional Eating Inventory [25] and the Anticipated Effects of Food Scale [16]. The items from the Emotional Eating Inventory emphasize eating behavior, while those from the Anticipated Effects of Food Scale focus on either junk food or healthy food in general. Items from the Emotion Eating Inventory were drawn from the following subscales: Eating Helps Manage Negative Affect (*Manage Negative Affect*), Eating Is Pleasurable and Useful as a Reward (*Pleasurable and Rewarding*), Eating Enhances Cognitive Competence (*Enhances Cognitive Competence*), and Eating Alleviates Boredom (*Alleviates Boredom*). Items from the Anticipated Effects of Food Scale were drawn from the Positive Feelings While Eating (*Positive Feelings*) subscale.

To better algin with our research aim, we modified these items from the Anticipated Effects of Food Scale [16] and the Emotional Eating Inventory [25]. Specifically, the original Anticipated Effects of Food Scale asked participants to report their expected emotional experiences when eating “JUNK food (e.g., sweets, salty snacks, fast foods, sugary drinks)” and the original Emotional Eating Inventory scale referred to people’s beliefs about the emotional effects of eating in general (e.g., “Eating helps me deal with sadness or emotional pain”). For both scales, we simply modified the wording of each statement to refer to the primary comfort food that participants had reported earlier so that the statements were specific to their comfort food.

Sample items include the following: “Eating [participant’s primary comfort food] seems to decrease my level of anxiety if I am feeling tense or stressed” (Manage Negative Affect subscale; 18 items, α = 0.91); “When I do something good, eating [participant’s primary comfort food] is a way to reward myself” (Pleasurable and Rewarding; 6 items, α = 0.80); “Eating [participant’s primary comfort food] helps me think and study better” (Enhances Cognitive Competence; 2 items, α = 0.80); “When I have nothing to do, eating [participant’s primary comfort food] helps me relieve the boredom” (Alleviates Boredom; 4 items, α = 0.84); and “While I am eating [participant’s primary comfort food], I feel Relieved” (Positive Feelings subscale; 15 items, α = 0.90). Participants rated each statement on a 7-point Likert scale, ranging from 1 (*Completely disagree*) to 7 (*Completely agree*).

***Frequency of Comfort Food Consumption.*** Because there is no established measure for the frequency of eating comfort foods, we developed a face-valid measure specifically for the present study. The frequency of participants’ comfort food consumption was measured in a number of ways, including questions regarding both the frequency of eating their primary comfort food and the frequency of eating any comfort food. The questions regarding frequency include the specific frequency in the short term (i.e., two weeks) and the general trend over the long term. To access the specific frequency of eating their primary comfort food and all comfort food in the short term, participants were asked “In the last two weeks, how many times have you eaten [participant’s primary comfort food] for psychological comfort?” and “Considering all your comfort foods (including but not limited to [participant’s primary comfort food]), how often have you eaten comfort food for psychological comfort in the last two weeks?” For both questions, participants responded with a whole number. To access the general frequency of eating their primary comfort food and all comfort food over the long term, participants were asked “How often do you typically eat [participant’s primary comfort food] for psychological comfort?” and “Considering all your comfort foods, how often do you eat comfort food for psychological comfort overall?”, with responses ranging from *Never* (0) to *Daily* (9).

***Participant characteristics.*** Participants reported their gender, age, height and weight (which were used to calculate their BMI), ethnicity, educational level, and social status. To evaluate social status, participants were asked to indicate their position on a social ladder, where the top represents those who are the best off in terms of wealth, education, and jobs, and the bottom represents those who are the worst off. Responses were made using a slide anchored by 0 (*Worst off*) to 100 (*Best off*) [26].

### 2.3. Statistical Analysis

Prior to conducting the main analyses, data were screened for normality and for the presence of outliers. A Kolmogorov–Smirnov test was performed, combined with the z-score of Skewness and Kurtosis, to determine whether the variables were normally distributed. All variables were normally distributed, except for the following: rating of Pleasurable and Rewarding subscale, frequency of eating primary comfort food in the last two weeks, and frequency of eating all comfort food in the last two weeks.

Outliers were defined using the Hoaglin and Iglewicz outlier labeling rule [27]. Eight outliers were identified on the variable “frequency of eating primary comfort food last two weeks”, and another eight outliers were identified on the variable of “frequency of eating all comfort foods last two weeks”. Removing these outliers had minimal impact on the results and, therefore, the analyses reported below included the full sample, with any differences in the results without the outliers noted in the text.

Descriptive analyses were conducted to summarize the type of primary comfort food that participants identified, the main reasons these foods were chosen as comfort foods, and the frequency of engaging in comfort eating. To assess gender differences in the frequency of eating comfort food, a Mann–Whitney U test was conducted. A chi-square test was conducted to examine the association between gender and comfort food preferences. Given the broad range of foods that were nominated as comfort foods, for ease of interpretation, this analysis used a dichotomous categorization of comfort foods as sweet (e.g., ice cream) vs. savory (e.g., pizza).

The next set of analyses examined participants’ expectations about comfort foods. Although the rating of Pleasurable and Rewarding subscale was not normally distributed, it did not exhibit severe deviations from normality. Given that *t*-tests and ANOVAs are robust to moderate violations of the normality assumption [28,29], we proceeded with parametric tests for consistency across analyses. First, to assess the extent to which participants expected potential benefits of eating comfort food, one-sample *t*-tests were conducted to compare ratings on each expectation subscale to the mid-point of the 7-point scale (i.e., a rating of 4). Scores above the mid-point indicate that participants expect benefits from eating their comfort food; scores below the mid-point indicate that participants do not expect benefits from eating their comfort food. Next, a repeated-measures ANOVA was conducted to test whether the ratings of the five expectation-related subscales differed significantly from each other. Finally, independent-samples *t*-tests were conducted to examine gender differences in ratings on the expectation subscales.

The last set of analyses examined the association between expectations about comfort foods and frequency of eating comfort foods. First, bivariate correlations were conducted to examine the individual relationship between each expectation subscale and frequency of eating comfort food. Although we preregistered that we would use Pearson correlation, Spearman correlation was more appropriate because the frequency variables were either count (consumption in the last two weeks) or ordinal (general frequency of consumption) data and because most of the frequency variables were not normally distributed. (Note that the pattern of results was similar when conducted using Pearson correlations.) Next, to examine the predictive relationships between the expectation-related variables and the frequency-related outcomes, a series of multiple regression analyses were conducted. Given that frequency variables of “Primary comfort food in the last two weeks” and “All comfort food in the last two weeks” (whether or not outliers were excluded) were not normally distributed, we applied a log transformation to normalize the data and the regression models were run with the log-transformed variables as the outcomes. In each analysis, all five subscales of expectations were entered simultaneously as predictors, and each frequency variable was examined separately as a dependent variable.

The study method and data analysis plan were preregistered on AsPredicted (https://aspredicted.org/3qgr-st49.pdf (accessed on 19 November 2024)).

## 3. Results

### 3.1. Primary Comfort Food

Due to the open-ended nature of the responses, a broad range of descriptions/terms were provided for participants’ primary comfort food. For some foods (e.g., chocolate, pizza, ice cream, burgers), the open-ended responses were quite uniform across participants. For other foods, the responses were quite varied and we decided to consolidate these responses into groups of similar foods, including the following: biscuits or cookies; chips or crisps; chicken dishes (e.g., chicken soup, chicken nuggets); potato dishes (e.g., hash brown, jacket potato); noodles or pasta (e.g., pho, lasagna); sweet bakery (e.g., doughnuts, cake); bread or toast (e.g., grilled cheese on toast); or others (foods that were not reported frequently enough to warrant a separate group). As shown in Table 1, participants most frequently identified chocolate as their primary comfort food, followed by chips or crisps and sweet bakery products. We categorized comfort foods reported by participants into sweet and savory to examine potential gender differences in food preferences. Foods that are ambiguous (e.g., McDonald’s) or can be either savory or sweet (e.g., popcorn) were categorized as “other.” Examples of the categorization are shown in Table 1. Savory foods accounted for 50% (107/214), while sweet foods accounted for 44.4% (95/214). The Pearson chi-square test showed that there was no significant association between gender and preferences for the comfort food category (sweet vs. savory), *χ^2^*(1) = 2.99, *p* = 0.084.

Participants were also asked to indicate the main reasons why they chose the food as their primary comfort food. They were allowed to select multiple reasons. The frequencies and percentages of each reason are shown in Table 2. The top three most commonly endorsed reasons were as follows: “it’s the comfort food I eat most often”; “it’s the easiest comfort food for me to obtain”; and “it comforts me in various situations or moods”.

### 3.2. Frequency of Eating Comfort Food

Most participants (*n* = 181; 84.58%) indicated that they ate their primary comfort food for psychological comfort at least once in the past two weeks. The reported frequency ranged from 0 to 20 times, with a mean of 2.89 (*SD* = 3.41; median = 2). Similarly, most participants (*n* = 201; 93.93%) indicated that they ate *any* comfort food for psychological comfort at least once in the past two weeks. The reported frequency ranged from 0 to 48 times, with a mean of 6.38 (*SD* = 7.12; median = 4).

Participants’ typical frequencies of eating their primary and all comfort foods are shown in Table 3. Most participants (*n* = 62; 28.97%) indicated that they ate their primary comfort food “About once a month”, with a mean of 5.80 (*SD* = 1.54; median = 6). Regarding the frequency of eating all comfort foods, the most frequently selected option (*n* = 63; 29.44%) was “Several times per week”, with a mean of 6.40 (*SD* = 1.67; median = 6).

The results of the Mann–Whitney U test indicated that there were no gender differences across all variables related to eating frequency of comfort food (smallest *p* = 0.178).

### 3.3. Ratings on Expectation Subscales

**Figure 1:**
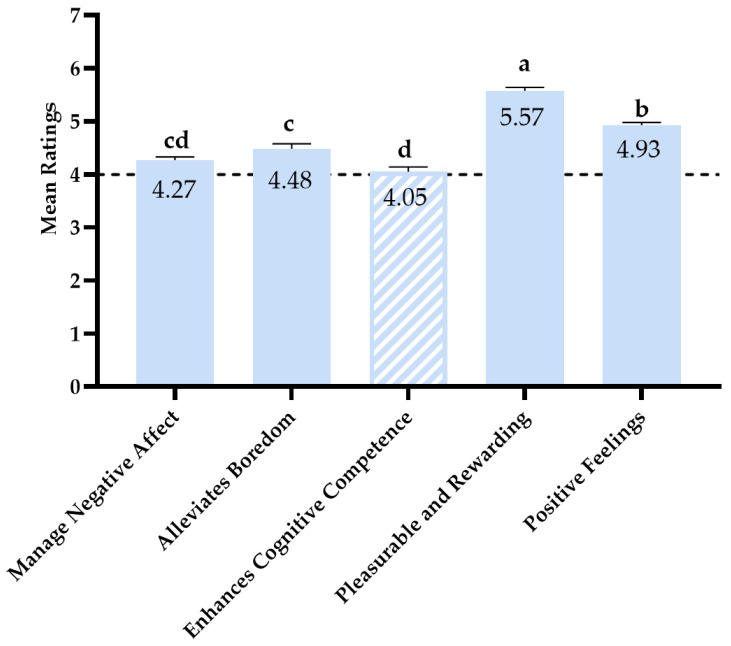
Mean ratings of expectation subscales. *Note.* Error bars represent standard errors. The horizontal dashed line represents the mid-point of the rating scale. Solid bars are significantly higher than the mid-point of the scale (*p* < 0.001); the striped bar is not significantly different from the mid-point. Bars with different superscripts are significantly different from each other (*p* < 0.001, except c vs. d: *p* = 0.004).

As displayed in Figure 1, one-sample *t*-tests revealed that mean ratings for all expectation subscales, except for the Enhance Cognitive Competence subscale (*p* = 0.600), were significantly higher than the mid-point of the scale (all *p*s < 0.001), indicating that participants believed that eating their comfort food confers positive benefits.

For the repeated-measures ANOVA, Mauchly’s test indicated that the assumption of sphericity was violated, *χ*^2^ (9) = 196.44, *p* < 0.001. Therefore, degrees of freedom were corrected using Greenhouse–Geisser estimates of sphericity (*ε* = 0.73). The results showed that mean ratings differed across the expectation subscales, *F*(2.91, 618.70) = 88.46, *p* < 0.001, $\eta_{p}^{2}$ = 0.293. Participants rated the Pleasurable Rewarding subscale as the highest, which was significantly higher than the other four subscales (*p*s < 0.001), followed by the Positive Feelings subscale, which was significantly higher than the remaining three subscales (*p*s < 0.001). The Alleviates Boredom subscale was rated significantly higher than the Enhance Cognitive Competence subscale (*p* = 0.004). The differences between Manage Negative Affect and Alleviates Boredom, as well as between Manage Negative Affect and Enhances Cognitive Competence, were not significant (*p*s > 0.15).

Some gender differences were observed regarding expectations about comfort foods. Compared to men, women scored higher on the Manage Negative Affect subscale as well as on the Alleviates Boredom subscale (see Table 4).

### 3.4. Correlation

Table 5 presents the Spearman bivariate correlations between expectation-related variables and frequency-related variables. Notably, the Manage Negative Affect subscale was significantly and positively correlated with all the frequency-related variables, as were Alleviates Boredom and Enhances Cognitive Competence. The Positive Feelings subscale was significantly and positively correlated with all frequency variables, except for primary comfort foods in the last two weeks. However, this result pattern was not robust: after excluding outliers on “All comfort food in the last two weeks”, the correlation between the Positive Feelings subscale and “All comfort food in the last two weeks” was no longer significant. The Pleasurable and Rewarding subscale was not significantly correlated with any of the frequency-related variables.

### 3.5. Regression

We next examined expectation-related variables as predictors of the frequency of eating comfort food (see Table 6). For the frequency of eating primary comfort food in the last two weeks, Alleviates Boredom, Enhances Cognitive Competence, and Pleasurable and Rewarding were significant independent predictors, with Pleasurable and Rewarding being the only predictor showing a negative association. When outliers were excluded, Alleviates Boredom and Enhances Cognitive Competence remained significant independent predictors, whereas Pleasurable and Rewarding was no longer a significant predictor. For frequency of eating any comfort food in the last two weeks, Manage Negative Affect and Alleviates Boredom were significant independent predictors. When outliers were excluded, Alleviates Boredom became the only significant independent predictor. For the typical frequency of eating primary comfort food, Alleviates Boredom, Enhances Cognitive Competence, and Pleasurable and Rewarding were significant independent predictors. For the typical frequency of eating all comfort food, Alleviates Boredom was the only significant independent predictor.

## 4. Discussion

People regularly consume comfort food, but relatively little is known about why they turn to comfort foods. Therefore, the present study had two main aims: First, to examine people’s expectations regarding their comfort food; second, to investigate whether these expectations are associated with their frequency of comfort eating.

Participants in this study endorsed a range of expected benefits from eating their primary comfort food. Specifically, ratings for the expectation subscales Pleasurable and Rewarding, Positive Feelings, Alleviate Boredom, and Manage Negative Affect were all significantly higher than the neutral mid-point (*Neither agree nor disagree*) of the scale. (Ratings for Enhances Cognitive Competence did not differ from the neutral mid-point of the scale.) Although participants endorsed a range of expected benefits of consuming comfort foods, the Pleasurable and Rewarding subscale received the highest endorsement overall, followed by the Positive Feelings subscale. These findings suggest that people believe they consume comfort food primarily for the positive reinforcements of rewarding themselves or gaining positive feelings while eating, rather than for the negative reinforcement of alleviating negative affect or boredom.

Participants’ expectations about the benefits of comfort foods may derive from various sources, including common cultural beliefs or how comfort food is depicted in the media. For example, ice cream is often depicted in films as a comfort food that people turn to after a breakup, and chicken soup is commonly seen as a comforting dish in Western culture. These expectations may also be shaped by personal memories related to food, such as the food they had in childhood or during momentous life events. Some research has identified “nostalgia food” as a subtype of comfort food (e.g., [6,30]), which carries fond memories from the past and relationships with others (e.g., partners, family, or friends [30,31,32]), and cultural traditions can also influence the food choices in these nostalgic scenes (e.g., [6]). Expectations regarding comfort foods might also stem from people’s own previous positive experiences with consuming comfort foods. For example, some research has shown that consuming a comfort food led to mood recovery following a negative mood induction (e.g., [7]). In addition, these expectations might also be related to physiological reactions that occur after consuming comfort food. For example, consuming foods rich in tryptophan (such as chicken) can increase serotonin, thereby improving mood states [33]. Some researchers have also suggested that comfort eating might be able to inhibit the activity of chronic stress response network [34]. These biochemical reactions could potentially shape individuals’ expectations about comfort food.

One of the reasons why it is important to understand people’s expectations about comfort foods is that expectations about the outcomes of a behavior can influence the likelihood of engaging in that behavior (e.g., [15]). Indeed, we found that participants’ expectations about the benefits of eating comfort foods were associated with their reported frequency of eating comfort foods. Specifically, Manage Negative Affect, Alleviates Boredom, and Enhances Cognitive Competence were positively associated with all frequency-related variables, with Alleviates Boredom showing the most consistent pattern of associations across both the correlational and regression analyses. The finding related to expectations about managing negative affect is in line with prior research showing that people seek comfort food when experiencing negative emotions (e.g., [6]). The findings that expectations about alleviating boredom are associated with the frequency of eating comfort food are consistent with experimental studies showing that participants in boring conditions consumed more chocolate and crackers than participants under neutral conditions [35,36], as well as with an ecological momentary assessment study showing that people snack more in their daily lives after experiencing stronger boredom [37]. As for the connection between Enhancing Cognitive Competence and frequency of eating comfort food, it might be that individuals tend to seek an extra energy boost to complete cognitive tasks, or perhaps even that cognitive tasks are related to stress and that consuming comfort foods helps alleviate that stress, indirectly boosting performance. Consistent with that interpretation, a field study has shown that students awaiting an exam reported a greater tendency to regulate their stress through eating [38].

It is interesting to note that the correlational and regression results between the expectation subscales and frequency of consuming comfort food showed a different pattern than participants’ subjective ratings of expectations did. That is, the Pleasurable and Rewarding subscale was the highest-rated subscale overall, but it was not significantly correlated with any of the frequency-related variables. It appeared only once as a significant negative predictor in the regression model; however, this effect disappeared after excluding outliers. Similarly, Positive Feelings was the next highest-rated subscale, but it was only weakly associated with the frequency-related variables in the bivariate correlations and was not a significant predictor in any of the regression models. It may be that participants’ ratings of each expectation subscale reflects what they hope to gain from eating comfort food, whereas the correlation relationship between the expectation-related variables and frequency-related variables may illustrate which expectation may motivate them to engage in actual behaviors. In other words, the inconsistent results may indicate a discrepancy between people’s beliefs about the benefits of consuming comfort food and the actual drivers of their behavior.

Another reason why it is important to understand people’s expectations regarding comfort food is that research has found that people’s expectations about the outcomes of a behavior can affect their actual experience. For example, in the area of nocebo effects, individuals could experience side effects of the treatment, partly because they expect to have negative consequences [39]. In other research, patients’ expectations about their myocardial infarction were associated with several functional outcomes and activities during the recovery stage at three- and six-month follow-ups, such as attendance at rehabilitation courses, time taken to return to work, and sexual dysfunction [40]. Similarly, it might be that people who expect to feel better after eating comfort foods are going to experience the greatest psychological benefits, which in turn could further increase their expectations of receiving benefits. Although previous research has provided inconsistent evidence about whether consuming comfort food actually has psychological benefits (e.g., [9,41]), it might be the expectations rather than the actual effect of comfort food shapes that comfort eating behavior. This is a question that should be addressed in future research.

In this study, we also explored potential gender differences related to comfort foods, but we found no significant gender differences in preferences for sweet or savory comfort food and no significant gender differences in consumption frequency of comfort food. Gender differences in comfort food preferences have been discussed in previous research, with one study suggesting that women were more prone to choose snack-related foods such as chocolate and ice cream for comfort, whereas men preferred meal-related foods, such as pizza, steak, burgers, and soups [2]. Another survey study found that the likelihood of women eating chocolate as comfort food under stress was three times higher than it was for men [42]. Given the inconsistent findings across studies, future research is needed to determine whether there are meaningful differences in the comfort food habits of women and men.

There are some limitations to the current study that need to be considered. First, the current study focused exclusively on exploring the expectations regarding participants’ primary comfort food. This was implemented so that questions about expectations could specifically target their own personal comfort food, enabling participants to imagine more vividly the scenarios while evaluating the expectations towards their comfort foods. However, it is possible that participants have different comfort foods that they associate with different expectations and functions. In addition, by focusing only on their primary comfort food, this study did not capture individuals’ general expectations regarding all comfort foods. Thus, future research could further examine differences in participants’ expectations towards different comfort foods or explore participants’ general expectation regarding their overall comfort foods. In addition, the measures of expectations regarding comfort foods were modified versions of existing measures, and the measure of the frequency of comfort food consumption was developed specifically for this study, and thus their validity and reliability have not been established beyond the current sample. Another limitation of this study is that our measures of consumption frequency were based on self-report data. Although these retrospective measures may reflect individuals’ perspectives based on their memory and subjective feelings, they may not be able to predict actual food intake in specific situations. Future studies examining consumption of comfort foods in specific settings would be beneficial in narrowing the gap in our understanding of how people’s expectations influence their consumption of comfort foods. In addition, given that the inclusion criteria required participants to eat comfort food at least occasionally, the frequency data may not be representative of the general population’s eating behaviors. Furthermore, participants in this study were not asked about the presence of eating disorders, so it is unclear to what extent these findings apply to individuals with problematic eating behaviors. It would be worth investigating the frequency of comfort eating and expectations in different populations to extend our understanding of the role of comfort food in daily life.

## 5. Conclusions

In conclusion, the present study provides insights into individuals’ expectations regarding comfort food and illustrates the relationship between expectations and the frequency of comfort eating. Specifically, we found that people expect to reward themselves or experience positive feelings through comfort eating, whereas it is the expectations of managing negative affect, alleviating boredom, and enhancing cognitive competence that are associated with their frequency of eating comfort food. By identifying the psychological benefits people expect to gain from comfort food, these findings may help develop interventions to address unhealthy comfort eating behaviors. Future research could further examine people’s comfort food choices and corresponding expectations under various scenarios (e.g., during celebrations, sadness, and boredom), and investigate which expectations could predict comfort eating and how this varies across scenarios, which would provide a richer understanding of how expectations shape consumption of comfort foods.

## Figures and Tables

**Table 1:** Top 10 most frequently mentioned comfort foods.

Rank	Primary Comfort Food	Category	Frequency
1	chocolate	Sweet	52
2	chips or crisps	Savory	35
3	sweet bakery	Sweet	17
4	pizza	Savory	15
5	noodles or pasta	Savory	13
6	ice cream	Sweet	12
7	chicken dishes	Savory	10
8	biscuits or cookies	Sweet	8
9	bread or toast	Savory	7
9	cheese	Savory	7
9	potato dishes	Savory	7
10	burger	Savory	4

**Table 2:** Reasons for choosing primary comfort foods.

Reasons	Frequency	Percentage
It’s the comfort food I eat most often.	169	78.97%
It’s the easiest comfort food for me to obtain.	101	47.20%
It comforts me in various situations or moods.	81	37.85%
It provides me with the strongest sense of comfort.	70	32.71%
It has the strongest sentimental meaning in my life.	45	21.03%
It’s deeply rooted in my cultural or family traditions.	29	13.55%
The comforting effect of this food lasts the longest.	25	11.68%
Other	17	7.94%

*Note.* Frequency refers to the number of times each reason was selected across all participants. Percentage indicates the proportion of participants (*n* = 214) who selected each reason. Because participants were able to select multiple reasons, the total percentage sums to >100%.

**Table 3:** Typical frequency of eating primary and all comfort foods.

Option	Primary Comfort Food		All Comfort Foods	
	Number	Percentage	Number	Percentage
Several times in your life	7	3.27	7	3.27
About once a year	3	1.40	3	1.40
Several times per year	27	12.62	17	7.94
About once a month	62	28.97	29	13.55
Several times per month	46	21.50	61	28.50
About once a week	31	14.49	21	9.81
Several times per week	34	15.89	63	29.44
Daily	4	1.87	13	6.07

*Note.* Participants were excluded from the study if they reported never eating comfort foods, so there are no responses of *Never* for these measures. Percentages are based on the total sample (*N* = 214).

**Table 4:** Gender differences in expectation subscale scores.

Subscale	Men (*M*, *SD*/*Mdn*)	Women (*M*, *SD*/*Mdn*)	*t*-Value	*p*-Value	Effect Size (*r*)
Manage Negative Affect	*M* = 4.11, *SD* = 1.02	*M* = 4.43, *SD* = 0.82	*t*(200.20) = −2.52	*p* = 0.013	*r* = 0.175
Alleviates Boredom	*M* = 4.30, *SD* = 1.34	*M* = 4.68, *SD* = 1.44	*t*(211) = −1.985	*p* = 0.048	*r* = 0.136
Enhances Cognitive Competence	*M* = 4.03, *SD* = 1.38	*M* = 4.07, *SD* = 1.37	*t*(211) = −0.222	*p* = 0.825	*r* = 0.015
Positive Feelings	*M* = 4.99, *SD* = 0.80	*M* = 4.87, *SD* = 0.76	*t*(211) = 1.151	*p* = 0.251	*r* = 0.079
Pleasurable and Rewarding	*M* = 5.45, *SD* = 0.96	*M* = 5.69, *SD* = 0.93	*t*(211) = −1.860	*p* = 0.064	*r* = 0.127

**Table 5:** Bivariate correlations between frequency-related variables and expectation-related variables.

	Primary Comfort Food in the Last Two Weeks	Typical Frequency of Primary Comfort Food	All Comfort Food in the Last Two Weeks	Typical Frequency of All Comfort Food
Manage Negative Affect	0.245 ***	0.270 ***	0.273 ***	0.265 ***
Alleviates Boredom	0.378 ***	0.355 ***	0.275 ***	0.268 ***
Enhances Cognitive Competence	0.320 ***	0.306 ***	0.145 *	0.143 *
Pleasurable and Rewarding	0.010	0.033	0.102	0.090
Positive Feelings	0.108	0.148 *	0.146 *	0.150 *

*Note.* * *p* < 0.05, *** *p* < 0.001.

**Table 6:** Regression coefficients predicting comfort eating frequency from all expectations-related variables.

Subscale	*β*	*t*	*p*
Primary comfort food in the last two weeks (log transformed)			
(*F*(5, 208) = 11.03, *p* < 0.001, Adjusted *R^2^* = 0.19)			
Manage Negative Affect	0.14	1.76	0.080
Alleviates Boredom	0.29	4.21	0.000
Enhances Cognitive Competence	0.24	3.18	0.002
Pleasurable and Rewarding	−0.15	−2.11	0.036
Positive Feelings	−0.06	−0.71	0.479
Primary comfort food in the last two weeks (outliers excluded, log transformed)			
(*F*(5, 200) = 8.84, *p* < 0.001, Adjusted *R^2^* = 0.16)			
Manage Negative Affect	0.10	1.16	0.248
Alleviates Boredom	0.27	3.80	0.000
Enhances Cognitive Competence	0.27	3.45	0.001
Pleasurable and Rewarding	−0.13	−1.76	0.080
Positive Feelings	−0.11	−1.21	0.229
All comfort food in the last two weeks (log transformed)			
(*F*(5, 208) = 5.43, *p* < 0.001, Adjusted *R^2^* = 0.09)			
Manage Negative Affect	0.21	2.48	0.014
Alleviates Boredom	0.20	2.76	0.006
Enhances Cognitive Competence	0.01	0.09	0.932
Pleasurable and Rewarding	−0.07	−0.91	0.364
Positive Feelings	0.02	0.22	0.828
All comfort food in the last two weeks (outliers excluded, log transformed)			
(*F*(5, 200) = 5.11, *p* < 0.001, Adjusted *R^2^* = 0.09)			
Manage Negative Affect	0.14	1.59	0.113
Alleviates Boredom	0.26	3.47	0.001
Enhances Cognitive Competence	0.03	0.34	0.732
Pleasurable and Rewarding	−0.05	−0.62	0.538
Positive Feelings	−0.01	−0.08	0.933
Typical frequency of primary comfort food			
(*F*(5, 208) = 9.67, *p* < 0.001, Adjusted *R^2^* = 0.17)			
Manage Negative Affect	0.13	1.54	0.125
Alleviates Boredom	0.26	3.71	0.003
Enhances Cognitive Competence	0.23	2.98	0.003
Pleasurable and Rewarding	−0.14	−1.96	0.051
Positive Feelings	0.00	0.03	0.978
Typical frequency of all comfort food			
(*F*(5, 208) = 4.70, *p* < 0.001, Adjusted *R^2^* = 0.08)			
Manage Negative Affect	0.16	1.88	0.061
Alleviates Boredom	0.20	2.68	0.008
Enhances Cognitive Competence	−0.01	−0.11	0.917
Pleasurable and Rewarding	−0.08	−1.08	0.283
Positive Feelings	0.09	0.97	0.332

## Data Availability

The raw data supporting the conclusions of this article will be made available by the authors on request.

## References

[1] Spence C. (2017). Comfort food: A review. Int. J. Gastron. Food Sci..

[2] Wansink B., Cheney M.M., Chan N. (2003). Exploring comfort food preferences across age and gender. Physiol. Behav..

[3] Dubé L., LeBel J.L., Lu J. (2005). Affect asymmetry and comfort food consumption. Physiol. Behav..

[4] Finch L.E., Tomiyama A.J. (2015). Comfort eating, psychological stress, and depressive symptoms in young adult women. Appetite.

[5] Wagner H.S., Ahlstrom B., Redden J.P., Vickers Z., Mann T. (2014). The myth of comfort food. Health Psychol..

[6] Soffin M.T., Batsell W.R. (2019). Towards a situational taxonomy of comfort foods: A retrospective analysis. Appetite.

[7] Macht M., Mueller J. (2007). Immediate effects of chocolate on experimentally induced mood states. Appetite.

[8] Standen E.C., Finch L.E., Tiongco-Hofschneider L., Schopp E., Lee K.M., Parker J.E., Bamishigbin O.N., Tomiyama A.J. (2022). Healthy versus unhealthy comfort eating for psychophysiological stress recovery in low-income Black and Latinx adults. Appetite.

[9] Finch L.E., Cummings J.R., Tomiyama A.J. (2019). Cookie or clementine? Psychophysiological stress reactivity and recovery after eating healthy and unhealthy comfort foods. Psychoneuroendocrinology.

[10] Privitera G.J., Welling D., Tejada G., Sweazy N., Cuifolo K.N., King-Shepard Q.W., Doraiswamy P.M. (2018). No calorie comfort: Viewing and drawing “comfort foods” similarly augment positive mood for those with depression. J. Health Psychol..

[11] Troisi J.D., Gabriel S. (2011). Chicken soup really is good for the soul: “Comfort food” fulfills the need to belong. Psychol. Sci..

[12] Goubet K.E., Chrysikou E.G. (2019). Emotion regulation flexibility: Gender differences in context sensitivity and repertoire. Front. Psychol..

[13] Zlomke K.R., Hahn K.S. (2010). Cognitive emotion regulation strategies: Gender differences and associations to worry. Personal. Individ. Differ..

[14] Flynn J.J., Hollenstein T., Mackey A. (2010). The effect of suppressing and not accepting emotions on depressive symptoms: Is suppression different for men and women?. Personal. Individ. Differ..

[15] Goldman M.S., Del Boca F.K., Darkes J. (1999). Alcohol expectancy theory: The application of cognitive neuroscience. Psychological Theories of Drinking and Alcoholism.

[16] Cummings J.R., Joyner M.A., Gearhardt A.N. (2020). Development and preliminary validation of the Anticipated Effects of Food Scale. Psychol. Addict. Behav..

[17] Montes K.S., Witkiewitz K., Pearson M.R., Leventhal A.M. (2019). Alcohol, tobacco, and marijuana expectancies as predictors of substance use initiation in adolescence: A longitudinal examination. Psychol. Addict. Behav..

[18] Morean M.E., Corbin W.R., Treat T.A. (2012). The Anticipated Effects of Alcohol Scale: Development and psychometric evaluation of a novel assessment tool for measuring alcohol expectancies. Psychol. Assess..

[19] Sharkansky E.J., Finn P.R. (1998). Effects of outcome expectancies and disinhibition on ad lib alcohol consumption. J. Stud. Alcohol.

[20] McMahon J., Jones B.T., O’Donnell P. (1994). Comparing positive and negative alcohol expectancies in male and female social drinkers. Addict. Res..

[21] Creamer M.R., Delk J., Case K., Perry C.L., Harrell M.B. (2018). Positive outcome expectations and tobacco product use behaviors in youth. Subst. Use Misuse.

[22] Parker M.N., Wilkinson M.L., Hunt R.A., Ortiz A., Manasse S.M., Juarascio A.S. (2022). Eating expectancies and hedonic hunger among individuals with bulimia-spectrum eating disorders who plan binge-eating episodes. Int. J. Eat. Disord..

[23] Hayaki J., Free S. (2016). Positive and negative eating expectancies in disordered eating among women and men. Eat. Behav..

[24] Combs J.L., Smith G.T., Simmons J.R. (2011). Distinctions between two expectancies in the prediction of maladaptive eating behavior. Personal. Individ. Differ..

[25] Hohlstein L.A., Smith G.T., Atlas J.G. (1998). An application of expectancy theory to eating disorders: Development and validation of measures of eating and dieting expectancies. Psychol. Assess..

[26] Adler N.E., Epel E.S., Castellazzo G., Ickovics J.R. (2000). Relationship of subjective and objective social status with psychological and physiological functioning: Preliminary data in healthy, White women. Health Psychol..

[27] Hoaglin D.C., Iglewicz B. (1987). Fine-tuning some resistant rules for outlier labeling. J. Am. Stat. Assoc..

[28] Boneau C.A. (1960). The effects of violations of assumptions underlying the t test. Psychol. Bull..

[29] Schmider E., Ziegler M., Danay E., Beyer L., Bühner M. (2010). Is it really robust? Reinvestigating the robustness of ANOVA against violations of the normal distribution assumption. Methodol. Eur. J. Res. Methods Behav. Soc. Sci..

[30] Locher J.L., Yoels W.C., Donna M., van Ells J. (2005). Comfort foods: An exploratory journey into the social and emotional significance of food. Food Foodways.

[31] Hepper E.G., Wildschut T., Sedikides C., Ritchie T.D., Yung Y.F., Hansen N., Abakoumkin G., Arikan G., Cisek S.Z., Demassosso D.B. (2014). Pancultural nostalgia: Prototypical conceptions across cultures. Emotion.

[32] Zhou X., van Tilburg W.A.P., Mei D., Wildschut T., Sedikides C. (2019). Hungering for the past: Nostalgic food labels increase purchase intentions and actual consumption. Appetite.

[33] Hulsken S., Märtin A., Mohajeri M.H., Homberg J.R. (2013). Food-derived serotonergic modulators: Effects on mood and cognition. Nutr. Res. Rev..

[34] Dallman M.F., Pecoraro N., Akana S.F., La Fleur S.E., Gomez F., Houshyar H., Bell M.E., Bhatnagar S., Laugero K.D., Manalo S. (2003). Chronic stress and obesity: A new view of “comfort food”. Proc. Natl. Acad. Sci. USA.

[35] Abramson E.E., Stinson S.G. (1977). Boredom and eating in obese and non-obese individuals. Addict. Behav..

[36] Havermans R.C., Vancleef L., Kalamatianos A., Nederkoorn C. (2015). Eating and inflicting pain out of boredom. Appetite.

[37] Aulbach M.B., Bamberg C., Reichenberger J., Arend A.K., Blechert J. (2025). Taming “hanger” and falling prey to boredom-emotional and stress-eating in 801 healthy individuals using ecological momentary assessment. Appetite.

[38] Macht M., Haupt C., Ellgring H. (2005). The perceived function of eating is changed during examination stress: A field study. Eat. Behav..

[39] Rief W., Hofmann S.G., Nestoriuc Y. (2008). The power of expectation: Understanding the placebo and nocebo Phenomenon. Soc. Personal. Psychol. Compass.

[40] Petrie K.J., Weinman J., Sharpe N., Buckley J. (1996). Role of patients’ view of their illness in predicting return to work and functioning after myocardial infarction: Longitudinal study. BMJ.

[41] Ong L.S., H I.J., Leung A.K. (2015). Is comfort food really good for the soul? A replication of Troisi and Gabriel’s (2011) Study 2. Front. Psychol..

[42] Gemesi K., Holzmann S.L., Kaiser B., Wintergerst M., Lurz M., Groh G., Böhm M., Krcmar H., Gedrich K., Hauner H. (2022). Stress eating: An online survey of eating behaviours, comfort foods, and healthy food substitutes in German adults. BMC Public Health.

